# Computational Evaluation of Amorphous Carbon Coating for Durable Silicon Anodes for Lithium-Ion Batteries

**DOI:** 10.3390/nano5041654

**Published:** 2015-10-13

**Authors:** Jeongwoon Hwang, Jisoon Ihm, Kwang-Ryeol Lee, Seungchul Kim

**Affiliations:** 1 Department of Physics and Astronomy, Seoul National University, 1 Gwanak-ro, Gwanak-gu, Seoul 08826, Korea; E-Mails: jeong.str@gmail.com (J.H.); jihm@snu.ac.kr (J.I.); 2 Center for Computational Science, Korea Institute of Science and Technology, 5 Hwarang-ro 14-gil, Seongbuk-gu, Seoul 02792, Korea; E-Mail: krlee@kist.re.kr (K.-R.L.); 3 Department of Nanomaterials Science and Engineering, Korea University of Science and Technology, 217 Gajeong-ro Yuseong-gu, Deajeon 34113, Korea

**Keywords:** lithium ion batteries, carbon coating, silicon anodes, durability, density functional theory, molecular dynamics

## Abstract

We investigate the structural, mechanical, and electronic properties of graphite-like amorphous carbon coating on bulky silicon to examine whether it can improve the durability of the silicon anodes of lithium-ion batteries using molecular dynamics simulations and *ab-initio* electronic structure calculations. Structural models of carbon coating are constructed using molecular dynamics simulations of atomic carbon deposition with low incident energies (1–16 eV). As the incident energy decreases, the ratio of *sp*^2^ carbons increases, that of *sp*^3^ decreases, and the carbon films become more porous. The films prepared with very low incident energy contain lithium-ion conducting channels. Also, those films are electrically conductive to supplement the poor conductivity of silicon and can restore their structure after large deformation to accommodate the volume change during the operations. As a result of this study, we suggest that graphite-like porous carbon coating on silicon will extend the lifetime of the silicon anodes of lithium-ion batteries.

## 1. Introduction

A lithium-ion battery (LIB) has been one of the most popular rechargeable batteries for portable electronics and electric vehicles due to the high energy density and power [1]. With the ever-increasing demand for high performance batteries, *i.e.*, high energy density and power, researchers have put great importance on manufacturing materials with better lithium capacity. Recently, silicon-based anodes have attracted much attention due to a storage capacity that is 10 times more than current graphite-based anodes. Unlike graphite, however, the major obstacle to a real world application of silicon anodes is their poor durability, which is due to an excessive volume expansion that is as much as 300% after lithium intercalation and causes structural fractures and weakened contact with current collectors [2,3]. Therefore, the main challenge for silicon-based anode materials in the field of lithium-ion batteries is maintaining their integrity during charging-discharging cycles.

To meet the need for durable, high-performance LIBs, there have been remarkable efforts in properly designing anode materials that have improved stability and capacity. One of these efforts takes advantage of the size effects of nanostructuring that silicon structure is smaller than naturally developed cracks so that crack is not developed. Nano-sized structures such as Si nanowires [4], Si nanopillars [5], and well-tailored arrays on Si thin-film [6] are employed so that a crack cannot develop inside them, making it possible for them to accommodate large strain without pulverization. Another approach involves the use of carbon-silicon composites such as C-Si core-shell nanowires [7], nanocomposite granules [8,9,10], and Si nanoparticles encapsulated by a carbon shell [11,12] based on the fact that carbon experiences less structural stress during lithium cycling and can function as a mechanical support.

In the same vein, the carbon-coated silicon has been suggested, and it is thought to have several advantages over using bare silicon as anode materials [13,14,15,16,17,18,19,20,21]; the carbon coating improves electronic conductivity and the contact with the current collectors while also maintaining the structural stability. It also prevents silicon anodes from directly contacting with electrolytes and forming solid-electrolyte interfaces (SEI) that can be stabilized on the C-film surface [22]. Carbon coating can be achieved via various ways of depositions [15,17,18,19] or plasma coating on both bulky materials and nanoparticles [23,24]. Several techniques are used in the manufacturing of commercial products; thus, it is highly possible to develop technology for real world applications if the characteristics of target anodes are well defined.

Desirable properties of the coating have been discussed by M. Joe *et al.* [25]; first, it is structurally nano-porous to selectively deliver lithium ions between electrolytes and silicon electrodes while it prevents delivering of electrolyte molecules, second, it has good electric conductivity to supplement the poor conductivity of silicon, and third, it is mechanically flexible and stretchy to keep wrapping silicon even under huge volume change and to keep good contact with current collectors.

Amorphous carbon (*a*-C) films grown by the deposition method are mostly amorphous in character [26] and can be categorized into diamond-like carbon (DLC) and graphite-like carbon (GLC) depending on the population ratio of *sp*^3^-hybridized bonds to *sp*^2^-hybridized bonds [27]. It is known that *a*-C film is harder and more brittle if the *sp*^3^ to *sp*^2^ ratio is high [23]. The brittle nature is inappropriate for silicon anode coatings because it should maintain the aforementioned advantages after large expansion and contraction. While DLC shows brittle characteristics, GLC, which has locally fullerene-like microstructures where the *sp*^2^ clusters are cross-linked through *sp*^3^ or *sp*^1^ sites, is expected to show better flexibility for the purpose. Indeed, GLC is electrically conductive because it has piecewise *sp*^2^ carbon flakes.

In this study, we investigate whether GLC has all aforementioned desirable properties. We deposit single atomic carbons on a bulky silicon surface to generate computational model structures of carbon coating. Incident energy is used as the controlling parameter, and we focus only on low incident energy because GLC is grown under low incident energy [28]. The structure is characterized in terms of bonding populations and porosity, followed by the calculation of electronic structure. We also consider the structural evolution of films under gradual strain as having *sp*^2^-dominate characteristics is not sufficient for flexibility. The properties of GLC are evaluated in regard to ideal coating conditions, one by one. Our structural model of GLC is different from the usual hydrogenated-GLC because it contains no hydrogen; however, it can be a good model of dehydrogenated GLC. Through this study, we suggest that GLC is the carbon film which satisfies the desired properties for durable silicon anodes of lithium-ion batteries.

## 2. Results and Discussion

Amorphous carbon films are grown with varying incident energies of atomic carbon. Microstructures of the resulting films clearly show that the population of *sp*^3^-hybridized carbon increases with increasing incident energy, while the porosity decreases as the energetic carbon atoms hit the surface with great force, making it dense and smooth overall (Table 1). This result is in accord with the previous studies of Huang *et al.*, but they focused on high incident energies of several tens-of-eV [28,29].

The energy-dependent microstructures of the films are explored by investigating radial distribution functions (RDF) and the population of constituent atoms with a specific coordination number (CN) ranging from 1 to 4. In this analysis, the tolerance of the C-C bond length is given to be ~10% larger than the C–C bond length in diamond to consider the uneven spacing between constituent atoms in an amorphized film. The population of *sp*^2^-hybridized carbon atoms (CN = 3) decreases from approximately 69% to 65% (see Table 1) with increasing incident energy from 1 eV to 16 eV. The portion of *sp*^2^ carbon is always much higher than that of *sp*^3^ carbon within the interested energy range, while the density increased by 39% and caused decrease in porosity by reducing the population of atoms with low coordination number (CN = 1 or 2) and by increasing *sp*^3^ atoms. Above 4 eV of incident energy, the densification gets intensified and the local structure becomes far different from the fullerene-like *sp*^2^-hybridized structure, even though the dominant bonding characteristic is *sp*^2^-hybridization (See Table 1). The distinct change appears in the population of carbon atoms with a coordination number equal to 4.

**Figure 1:**
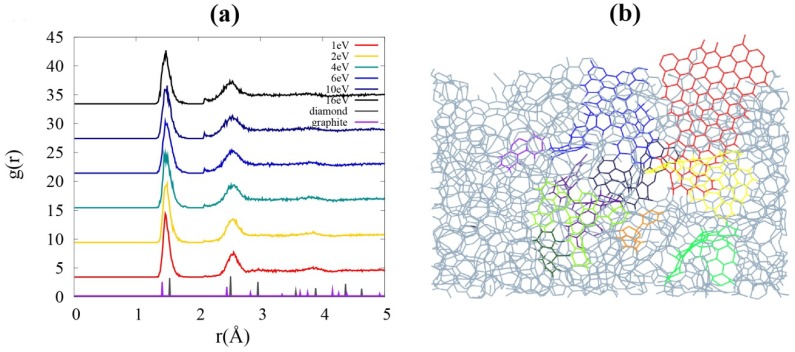
(**a**) Radial distribution functions (RDF) g(*r*) of the films with different incident energies. Two broadened peaks at higher energies imply the formation of *sp*^3^-hybridized carbons. (**b**) A sliced image of the film grown with incident energy of 1 eV. Basic structural units (BSUs), the graphene flakes, are embedded in the film. Flakes of different color codes are connected to each other by *sp*^1^ chains, *sp*^3^ intersections, or both.

RDF in Figure 1a indicates that there is only a short-ranged order in these amorphous systems. For comparison, the RDF of crystalline diamond and graphite are also drawn with the reduced peak heights. Due to the crystallinity, the peaks are essentially narrow and relate to the lattice sites. The first and second peaks in the RDF for the carbon films get broader and new small peaks appear between the two major peaks as the incident energy increases. This result implies that various bonding types, such as *sp*^1^, *sp*^2^, and *sp*^3^ hybridizations, coexist within the film under ambient conditions, where the ground state structure of crystalline carbon is graphite, owing to the impact energy of the incident carbon atom.

**Table 1:** Energy dependent bond population and density of the deposited films. In this incident energy range, *sp*^2^ hybridization with a coordination number equal to 2 is always dominant, whereas the *sp*^3^ population increases and *sp*^1^ decreases with increasing energy.

Energy (eV)	Coordination Number (%)				Density (g/cm^3^)
	1	2	3	4	
1	3.03	18.87	69.39	8.71	2.04
2	3.95	19.75	64.17	12.13	1.90
4	2.78	15.70	63.92	17.59	2.42
6	1.54	15.96	64.16	18.33	2.61
8	3.51	14.88	64.51	17.11	2.53
10	2.75	14.26	64.70	18.29	2.73
16	2.47	14.52	65.65	17.27	2.83

**Figure 2:**
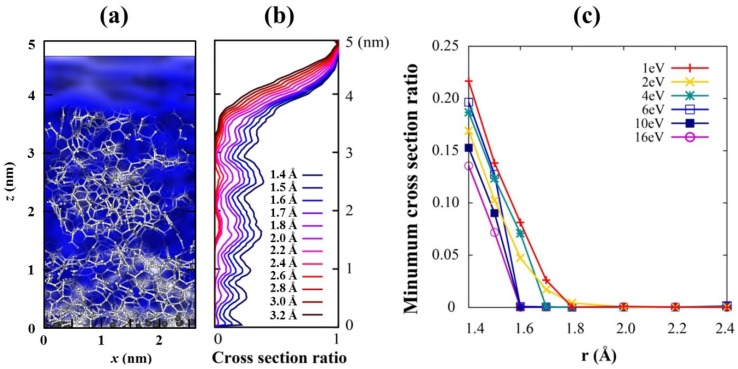
(**a**) Ionic channels with *r* =1.6 Å (blue cloud) through the film where Li ions will defuse along it. (**b**) The cross section ratio of the channels, which is the relative size of the Li channel compare to the total cross section, from the top surface of the carbon to the C/Si interface for 1 eV deposition energy with various *r*. 0 stands for complete closing of the channel, while 1 does complete opening of the channel. (**c**) Minimum value of the channel cross section ratios for films with various incident energies. The Li channel with *r* ≥ 1.6 Å closes for high incident energies above 4 eV.

A remarkable feature of a film grown at low incident energy (1 eV) is that the configuration can be described as connection of basic structural unit (BSU), as seen in Figure 1b. The whole structure is a very complicated mixture of chain and mesh, yet slices of the film well display the *sp*^2^ network embedded in it. An abundance of *sp*^2^ bonds offers less brittleness under stress [23] as well as the flexibility which comes with increased porosity. Moreover, we find that the film shows superior features for lithium diffusion in terms of sub-nano-porosity by comparing it to the films grown at higher energies (Figure 2).

Porosity and distribution of pores have been examined to check whether a selective conducting channel of lithium ions exists in the coating, which is the first one of the aforementioned three desirable conditions. We take account of 15 configurations among MD trajectories for all films to consider the thermal motions of the constituent atoms at room temperature. These motions can repeatedly modify a channel and affect concomitant ion conductivity. An ion conducting channel is defined as pores with a specific radius (*r* in Figure 2c) connected through the top surface of the film to the C/Si interface, in which any point is separated from all other atoms more than given radius *r*. This is conceptually similar to van der Waals surface but with different radii. Isolated pores in the film are excluded in the channel. The connected pores, represented by the blue cloud in Figure 2a, act as a channel through which Li ions can flow, *i.e.*, channels for Li intercalation.

Figure 2b shows that cross section of the channels, normalized by 1, generally decreases from the top surface of the carbon film deep into the C/Si interface for every channel radius. A cross section ratio of the channel at a given height *h* is defined by the area of the channel cross section relative to the whole film area. It becomes the minimum value 0 when the channel is completely closed and does the maximum value 1 when nothing blocks Li ions. The smaller the deposition energy is used, the larger the channel is generated (Figure 2c). A channel with a larger radius closes earlier, and only those of maximum 1.6–1.8 Å radius survive for very low incident energies. We speculate that the small size of the channel is due to the small carbon source (C_1_) we used; larger channels will exist if a bigger carbon source is used.

Molecules of electrolytes are much bigger than Li ions, so they need much larger channels to penetrate through the film. Therefore, if we can grow films with pores of a predetermined size, that is, large enough to conduct Li ion and small enough to block electrolyte molecules, the proposed film will play a role as a molecular sieve which offers conducting pathways only for Li ions. We assume that the minimum C–Li distance for Li conducting channel is 1.6 Å because it is the C–Li distance in Li-adsorbed graphene [30], and it is half of the interlayer distance of graphite, which is the most widely used anode material in current LIBs.

As Figure 2c shows, the minimum cross section, the bottleneck of ion flow, is almost closed for energies of higher than 4 eV and is open for lower energies. This is expected because a carbon film grown at higher incident energy is compact and has relatively small pores in it. This feature is also consistent with our density analysis tabulated in Table 1, where the density jumps to higher values as the incident energy increases from 2 eV to 4 eV. Therefore, low-energy incidence is the essential condition for carbon films to have Li-selective conducting channels. The maximum size of a desirable channel, *i.e.*, the largest channel that can block electrolyte molecules, is not discussed here because even the largest open channel (1.8 Å) is much smaller than any electrolyte molecule; for example, the sizes of ethylene carbonate (EC) and diethylene carbonate (DEC) are about 5–6 Å.

As the second desirable condition of the coating material is having good electric conductivity, we now move to the electronic property of the lowest energy case (1 eV) because it shows the best quality of an ionic conductor. The carbon film is found to be metallic from the density functional theory calculations, and its electronic conducting property can be explored through the degree of delocalization of the electronic wave functions near the Fermi energy; the more delocalization of wave functions, the higher electric conductivity.

A numerical wave function at *n*-th electronic state can be written as

(1) $$\Psi_{n}\left(\vec{r}\right)=\underset{i}{\sum }a_{i}\delta \left(\vec{r}-{\vec{r}}_{i}\right)$$

where |*a_i_*| is the modulus of the wave function at each grid point and the index *i* runs over all 3-dimensional grid points. Electron density from Ψ*_n_* in an infinitesimal volume centered at ${\vec{r}}_{i}$ is given to be |*a_i_*|^2^ for normalized Ψ*_n_.* We define delocality, the degree of delocalization of the function, of the *n*-th eigenstate as:

(2) $$D_{n}=\frac{{\left[\int^{\text{​}}|\Psi_{n}{|}^{2}\text{d}\vec{r}\right]}^{2}}{\int^{\text{​}}|\Psi_{n}{|}^{4}\text{d}\vec{r}}=\frac{\sum_{i}{\left|a_{i}\right|}^{2}∙\sum_{i}{\left|a_{i}\right|}^{2}}{\sum_{i}{\left|a_{i}\right|}^{4}}.$$

*D_n_* gives a minimum value 1 when the wave function is perfectly localized at one grid point and a maximum value of *N*, the total number of grid points, when the wave function is uniformly distributed over all grid points. If *a_i_* is a constant at *M* (0 < *M* < *N*), *i.e.*, |*a_i_*| = 1/*M*, grid points and zero at all other points, *D_n_* becomes *M*, which is the size of the space where the wave function is non-zero. Total *N* = 80 × 80 × 100 grid points is used in this calculation.

**Figure 3:**
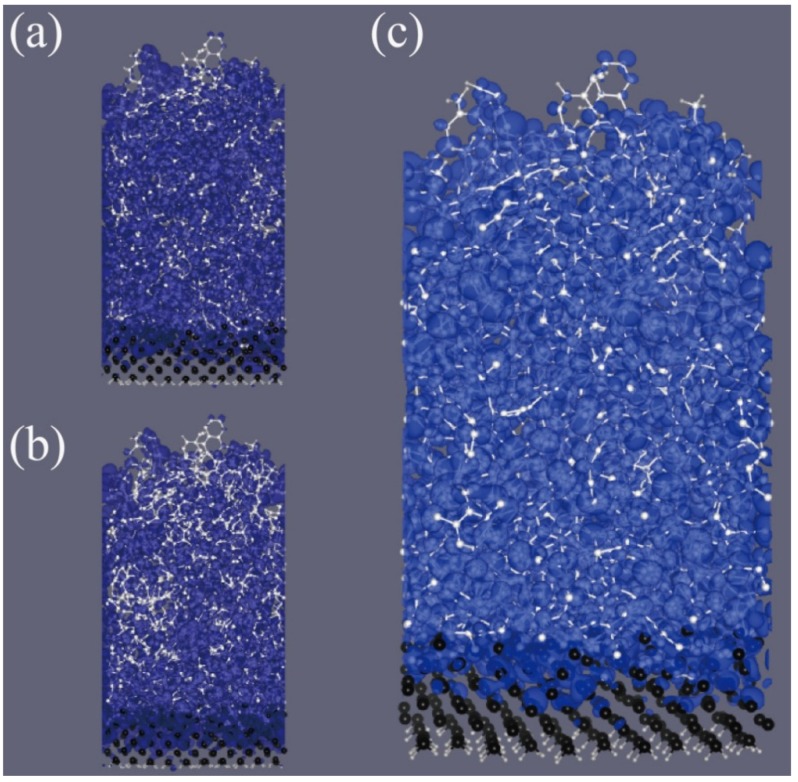
The moduli of wave function of (**a**) the highest occupied state and (**b**) the lowest unoccupied state of 1 eV-grown carbon film. Two wave functions are distributed in whole space of film. (**c**) The local density of states (LDOS) integrated over [$E_{\text{F}}-\;0.1\text{ eV}$, $E_{\text{F}}+0.1\text{ eV}$] energy range. The wave functions and the LDOS well display the delocalized character of electronic states near the Fermi level. White and black balls represent C and Si atoms, respectively. The silicon substrate is truncated and terminated by hydrogen atoms (represented by small gray balls at the bottom surface).

The wave function of the highest occupied state and the lowest unoccupied state are well delocalized (Figure 3a,b), which are chosen to show the delocalized character of individual electronic states near Fermi level. The resulting delocality of states near the Fermi level reaches approximately 17% of the π-state of graphene at the Dirac point, which is the maximally delocalized state of all carbon systems. Because the states at Dirac points of graphene give extraordinarily high conductivity, 17% of delocality might be large enough to improve the conductivity of silicon anodes significantly. The local density of states (LDOS), $\text{LDOS}\left(E,r\right)=\;\underset{E<\epsilon_{n}<E+\Delta E}{\sum }{\left|\Psi_{n}\left(r\right)\right|}^{2}$, near the Fermi level is similarly dispersed over the film. Figure 3c is the integrated LDOS for the *E*_F_ − 0.1 eV< *E* <*E*_F_ + 0.1 eV energy range to which 180 electronic states contribute. It is well dispersed over the film; therefore, the film can deliver many electrons between the electrolyte and silicon.

As the third desirable condition of the carbon coating is being flexible and stretchable, we conduct stress-strain simulations to check whether structural properties of carbon coating are intact after applying tensile and compressive biaxial strain gradually and the coating keeps wrapping during the expansion and contraction. This simulation is devised to mimic the situation of volume changes by charging and discharging. Applied strain is increased up to 50% in both the *x* and *y* directions, which corresponds to about 338% (1.5^3^ = 3.38) volume expansion and is then reduced back to 0%. During an expansion, the shapes of graphene flake, BSU, change little, but only inter-flake connection bonds change much, which causes less brittle character. It is worth to mention that the carbon coated silicon film is slightly stiffer than bare silicon film, which is a common property of amorphized films. The film goes through plastic deformation after a certain yield point (approximately 21% strain) when several linear *sp*^1^ bonds bridging *sp*^2^ carbon flakes are broken due to their vulnerability to stress, making them more easily broken compared to those atoms which consist of flakes. However, at the same time, reconstructions occur and new connections are made. Because the volume expansion due to lithiation occurs in a relatively long period of time compared to a time scale of atomic dynamics, the reconstruction will happen continuously during expansion.

**Figure 4:**
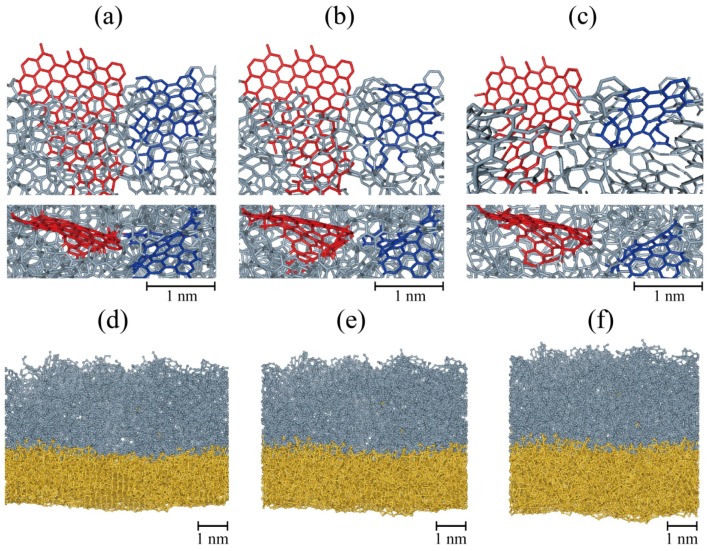
Structural evolution during expansion (**a**–**c**) and contraction simulations of the 1 eV-grown film. Biaxial strains are (**a**) 0%; (**b**) 12.5%; (**c**) 25%; (**d**) 35%; (**e**) 25%; and (**f**) 15%. The film is expanded from 0% to 50%, then returns to 0% strain. In (**a**–**c**), only a part of carbon film is shown to clearly display the structural evolution of BSUs colored in blue and red. In (**d**–**f**), the composite of carbon film (gray) and the silicon substrate (yellow) almost recover its original shape after a cycle of expansion and contraction.

While the applied stress is released through the breaking of linear bonds, the carbon film still covers the Si surface and shows stability. Figure 4a–c show the structural evolution of *sp*^2^ flakes embedded in the film during the expansion. In the as-grown film, two flakes (colored red and blue in Figure 4a) are connected and become separated with increasing strain (Figure 4c). *Sp*^2^ carbon flakes and pores are remained but are now corrugated or modified due to the locally random stress gradient caused by amorphous characteristics.

Once it was expanded by 50% in the planar directions, the film was contracted to its original size. The same magnitude of strain rate to the expansion simulation was used. Cracks which developed at a higher strain disappear at approximately 30% as a consequence of bond reformation between the constituent atoms, and the composite of the carbon film and the silicon substrate almost recovered its original shape (see Figure 4d–f). From these expansion and contraction simulations, we can assert that the film is able to endure almost reversibly.

We discuss properties of GLC coating for a longer lifespan of the silicon anodes of LIBs using the model structures generated by atomic deposition simulations of this study. Polymeric fullerene (C_60_) has also been suggested as another type of a desirable coating of carbon [25] in the same vein. Their common properties, *i.e.*, desirable properties for an ideal carbon coating, include being structurally sub-nano-porous (for selective Li ion conduction), having large portion of *sp*^2^ carbons (for electric conduction), and consisting of loosely connected BSUs (for good flexibility). There are many experimental techniques to fabricate various properties of carbon films, such as plasma deposition and chemical vapor deposition (CVD). For example, by using hydrocarbon plasma deposition, we can control both porosity (from almost non-pore [26] to micrometer-pore-sized structures [31]) and hardness (from very stiff [26] to flexible [32]). The electric conductivity of amorphous carbon film can be improved by dehydrogenation with thermal treatment. Experimentalist may try the low pressure plasma deposition with the small carbon precursors, such as C_2_H_2_ or CH_3_ (for sub-nano porosity) along with successive thermal treatment for dehydrogenation (for good electric conductivity). Therefore, we expect that a desirable carbon coating on silicon must be economically achievable by applying developed technologies. From the observations of this study and Joe *et al.* [25], the film can be grown with a deposition method by choosing a deposition energy that is just large enough energy to grow the film.

## 3. Methods

The process of carbon film growth is simulated by classical molecular dynamics (MD) simulations with Large-scale Atomic/Molecular Massively Parallel Simulator (LAMMPS) code [33]. Interactions between atoms are described by the Tersoff potential [34], which is a common choice for the system composed of carbon and silicon because it provides a successful description of all of the types of interactions among them. It is known that the Tersoff potential underestimates the portion of *sp*^3^ in amorphous carbon films, so we restrict our interest to low-energy deposition to reduce errors arising from the potential. Atomic carbons with definite energy are shot onto a silicon (111) substrate, which is 2.7 nm × 2.7 nm × 3.0 nm (23 total layers of silicon atoms) and has the periodic boundary condition in the lateral direction.

We perform independent simulations with various deposition energies ranging from 1 eV to 16 eV. For all cases, atomic configurations are updated every 0.25 fs; new carbon atoms are deposited every 3.75 ps until the total number of incident atoms becomes 3000. During the film-growth, all atomic motions in the reactive surface region (carbon atoms and ten atomic layers of silicon) are governed by the NVE ensemble, whereas the remaining part is controlled by NVT as a heat-bath. The four bottom layers were fixed to keep the bulk silicon structure. The heat-bath layers equilibrate the system to room temperature (300 K) by rescaling the translational velocities of affiliated atoms according to the Berendsen thermostat [35]. The thickness of the top free-layers, where NVE is applied, is determined by considering the penetration depth of energetic atomic carbons with the highest incident energy (16 eV) on free layers.

Electronic structures of as-deposited films are investigated by *ab initio* density functional calculations using Spanish Initiative for Electronic Simulations with Thousands of Atoms (SIESTA) code [36] with local density approximation (LDA) of the exchange correlation functional [37]. Split-valence double zeta (DZ) pseudo-atomic orbital (PAO) basis set is employed, and an energy cutoff for real-space charge density is set to be 100 Ry. For calculation efficiency, a silicon substrate was truncated off, leaving a few layers. Carbon and silicon atoms on both the top and bottom surfaces of the slab geometry are passivated by hydrogen atoms.

We investigate the mechanical strength of GLC and examine whether GLC retains its structure after expansion and contraction. A doubled supercell in two lateral directions (*i.e.*, 5.4 nm × 5.4 nm) was used for these straining simulations, and the applied strain rate was 0.001/ps. Accordingly, the length of the simulation box at time *t* takes the form *L*(*t*) = *L*_0_(1 + *et*), where *e* is the strain rate and *L*_0_ is the initial length.

## 4. Conclusions

We show that, using molecular dynamics simulations and *ab initio* electronic structure calculations, graphite-like carbon (GLC) coating grown at low deposition energy potentially lengthens the lifespan of the silicon anodes of lithium ion batteries (LIBs), as it is structurally porous, electrically conductive, and mechanically flexible. It is demonstrated that the GLC film recovers its physical properties after expansion and contraction, which implies good cyclability. However, the carbon film gradually degrades as the deposition energy increases and becomes denser when more *sp*^3^ carbon atoms are formed. We propose loosely connected BSUs as a fundamental requirement for durable carbon coating based on the fact that GLC can be characterized by such a structural unit and it may actually be grown successfully with a low energy deposition of the carbon source.
